# Lactic Acid Bacteria as Natural Antimicrobials: Biofilm Control in Food and Food Industry

**DOI:** 10.3390/antibiotics15030248

**Published:** 2026-02-27

**Authors:** Minji Kim, Jesmina Khatun, Fazlurrahman Khan, Young-Mog Kim

**Affiliations:** 1 Research Center for Marine Integrated Bionics Technology, Pukyong National University, Busan 48513, Republic of Korea; 2 Marine Integrated Biomedical Technology Center, The National Key Research Institutes in Universities, Pukyong National University, Busan 48513, Republic of Korea; 3 Department of Food Science and Technology, Pukyong National University, Busan 48513, Republic of Korea; 4 Ocean and Fisheries Development International Cooperation Institute, Pukyong National University, Busan 48513, Republic of Korea; 5 International Graduate Program of Fisheries Science, Pukyong National University, Busan 48513, Republic of Korea

**Keywords:** lactic acid bacteria, foodborne pathogens, biofilm control, nanoparticles, encapsulated formulations

## Abstract

Biofilm production by foodborne pathogens poses significant challenges to food safety and quality, leading to contamination, deterioration, and substantial economic losses for the food industry. Traditional biofilm control methods, such as chemical disinfectants, antibiotics, and preservatives, are sometimes ineffective against persistent biofilms, raising concerns about antimicrobial resistance and the accumulation of chemical residues. Lactic acid bacteria (LAB) have emerged as attractive natural biocontrol agents due to their ability to produce a wide range of antimicrobial secondary metabolites, including bacteriocins, organic acids, hydrogen peroxide, and biosurfactants. This paper thoroughly examines the effect of LAB and their metabolites in preventing and destroying biofilms generated by bacteria relevant to food systems, including *Listeria monocytogenes*, *Salmonella enterica*, *Escherichia coli*, and *Pseudomonas* spp. The processes causing LAB-mediated biofilm attenuation are thoroughly investigated, including competition for nutrients and adhesion sites, interference with quorum sensing (QS), and metabolic inhibition. Furthermore, recent breakthroughs in LAB-based techniques for food preservation and facility hygiene are discussed, including the creation of LAB-derived antimicrobial coatings, biosurfactant-based cleaning agents, and probiotic bio-coatings for industrial sanitation. The incorporation of nanotechnology has enhanced LAB applications by enabling the creation of LAB-mediated metallic nanoparticles and encapsulated formulations that improve metabolite stability and facilitate controlled release. The combination of LAB metabolites, natural preservatives, and eco-friendly materials in active packaging provides sustainable alternatives to synthetic chemicals. Overall, this review emphasizes the potential of LAB and their bioactive derivatives as environmentally friendly and practical tools for controlling biofilms and preserving food, thereby promoting safer food production systems and accelerating the food industry’s transition to green, sustainable technologies.

## 1. Introduction

Microbial biofilms pose a critical challenge in food processing environments, consisting of structured microbial communities embedded within extracellular polymeric substances (EPS) matrices that adhere to various surfaces [1,2]. These EPS matrices, composed of polysaccharides, proteins, nucleic acids, and water, enable surface attachment and protect bacteria from environmental stresses, UV radiation, extreme pH, and antimicrobial agents [3]. Biofilms pose significant threats to food safety by facilitating cross-contamination, harboring pathogenic and spoilage microorganisms, and demonstrating enhanced resistance to traditional disinfectants and sanitizers [4]. The persistence of biofilms in food processing facilities leads to equipment corrosion, impaired heat transfer, reduced shelf life, and increased risk of foodborne disease transmission [5]. Effective control requires integrated approaches combining mechanical cleaning, chemical sanitation, and novel technologies to address this persistent contamination source [6].

**Figure 1:**
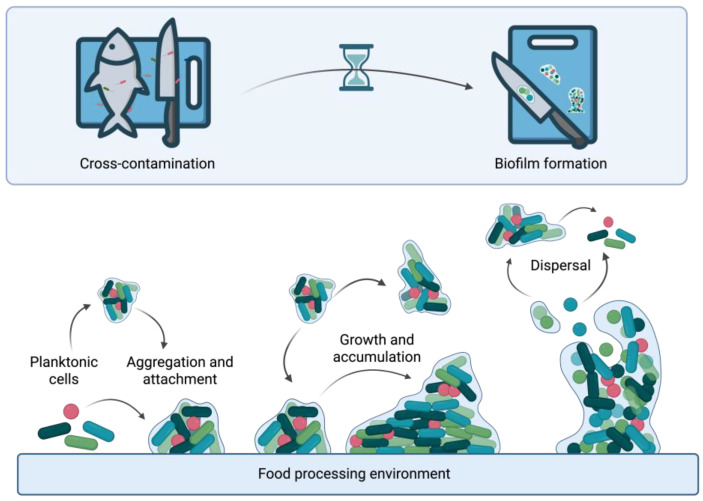
Biofilm formation in food processing environments. Planktonic cells aggregate and reversibly attach to a surface, initiating biofilm development. Cells then irreversibly adhere, mature into a structured community encased in extracellular matrix. Lastly, cells disperse to enable cross-contamination and colonize new sites, completing the cycle. Created in BioRender. Kim, M. (2026) https://www.biorender.com/ (accessed on 1 January 2026).

Bacterial biofilms are complex surface-attached communities embedded in self-produced EPS composed of polysaccharides, proteins, nucleic acids, and lipids [7,8]. Biofilm development progresses through distinct phases: reversible attachment, irreversible adhesion, EPS production, maturation, and dispersal [8,9]. During initial attachment, planktonic cells adhere through weak interactions, followed by irreversible adhesion mediated by bacterial adhesins [8]. Mature biofilms develop three-dimensional architectures resembling coral reefs with microcolonies, channels, and nutrient gradients [9,10]. The EPS matrix provides mechanical stability, mediates surface adhesion, and acts as an external digestive system [7]. Biofilm-associated cells exhibit dramatically increased antimicrobial resistance compared to planktonic counterparts [11]. Active dispersal completes the biofilm lifecycle (Figure 1), enabling colonization of new surfaces through specialized dispersal cells [12]. This multicellular organization presents significant challenges in healthcare and industrial settings [13].

Biofilm formation by foodborne pathogens on food-contact surfaces poses significant challenges in food processing environments. *Listeria monocytogenes* readily forms biofilms on various surfaces, including stainless steel, Teflon, nylon, and polyester floor sealant, with polyester showing the highest biofilm coverage [14]. These biofilms serve as persistent contamination sources, as microorganisms within biofilms demonstrate 10–1000 times greater resistance to antimicrobial agents compared to planktonic bacteria [15]. Key foodborne pathogens forming biofilms include *L. monocytogenes*, *Salmonella enterica*, *Escherichia coli*, *Staphylococcus aureus*, and *Pseudomonas aeruginosa* [16,17]. Persistent *L. monocytogenes* strains show enhanced surface adherence and biofilm formation capabilities, enabling survival for years in processing facilities [18]. Biofilm formation leads to cross-contamination, reduced shelf life, and increased foodborne illness risks [5,19]. Control strategies include mechanical cleaning, chemical sanitizers, thermal treatments, and emerging biological approaches [20].

Traditional biofilm control methods using chemical disinfectants, antibiotics, and preservatives face significant limitations, including antimicrobial resistance development, toxic residue concerns, and limited penetration through biofilm matrices [21,22]. These challenges have driven research toward sustainable, biologically derived alternatives. Natural control methods show promise, including bacteriocins, bacteriophages, essential oils, plant extracts, and enzymatic treatments [23,24]. Emerging strategies encompass nanotechnology-based approaches, quorum-sensing (QS) inhibition, antimicrobial photodynamic therapy, and ultrasonic systems [25,26]. Microbe-derived biological agents offer eco-friendly solutions to combat mono- and mixed-bacterial biofilms while avoiding the negative environmental and health impacts of conventional chemicals [27]. Combinatory approaches integrating multiple anti-biofilm strategies are increasingly recognized as necessary to enhance effectiveness and overcome biofilm resistance [24,28].

Lactic acid bacteria (LAB) are Gram-positive, non-sporulating, generally recognized as safe (GRAS) microorganisms that serve as promising natural biocontrol agents against foodborne pathogens [29]. LAB produce diverse antimicrobial metabolites, including organic acids (lactic, acetic), hydrogen peroxide, diacetyl, carbon dioxide, bacteriocins, reuterin, and biosurfactants that effectively inhibit pathogenic microorganisms [30,31]. These compounds demonstrate significant antibiofilm activity against major foodborne pathogens such as *L. monocytogenes*, *S. enterica*, *E. coli*, and *S. aureus* on food contact surfaces [32,33].

LAB employ multiple antimicrobial mechanisms that make them effective biocontrol agents in food systems. The primary mechanism involves organic acid production, which lowers environmental pH and creates hostile conditions for pathogens [34]. LAB also produces hydrogen peroxide, contributing to oxidative stress that damages cellular components [35,36]. Bacteriocins, ribosomally synthesized antimicrobial peptides, specifically target closely related bacteria by disrupting cell membranes [31,32]. These compounds demonstrate particular effectiveness against foodborne pathogens, including *L. monocytogenes*, *S. aureus*, and *E. coli* [32]. Additionally, LAB compete with pathogens for nutrients and adhesion sites, limiting their colonization ability [36]. These multifaceted antimicrobial properties position LAB as promising natural alternatives to chemical preservatives in food preservation [37].

Recent research has significantly expanded LAB applications beyond traditional fermentation to include innovative delivery systems and sustainable packaging solutions. LAB-derived metabolites, particularly bacteriocins and organic acids, are being incorporated into edible films and active packaging materials to extend food shelf life through antimicrobial activity [38,39]. These biodegradable films offer the dual benefits of environmental sustainability and effective pathogen control [40,41]. LAB exopolysaccharides demonstrate excellent film-forming properties for edible packaging applications while providing bioactive functions, including antioxidant and antimicrobial activities [39]. Advanced delivery systems utilize LAB as both passive encapsulation targets and active carriers through surface adsorption, intracellular loading, and co-encapsulation in hydrogels [42]. These bio-based approaches align with consumer demand for clean-label products and environmentally responsible technologies, supporting applications in functional foods, precision nutrition, and sustainable agriculture [43,44,45].

Furthermore, the LAB products have been extensively utilized to synthesize metal nanoparticles, including silver and gold, which exhibit potent antimicrobial and antibiofilm activities against pathogenic bacteria such as *P. aeruginosa* and *S. aureus* [46,47]. These biogenically synthesized nanoparticles effectively inhibit biofilm formation and eradicate established biofilms while reducing virulence factors [47]. LAB produce antimicrobial compounds like bacteriocins that demonstrate dose-dependent biofilm inhibition against foodborne pathogens, including *L. monocytogenes*, *S. aureus*, and *E. coli* [32]. LAB biofilms themselves possess antimicrobial properties against pathogenic microorganisms [48]. The combination of LAB metabolites with other antimicrobials creates synergistic effects, enhancing their antibiofilm activity [32,49]. These natural biopreservation strategies offer promising alternatives to conventional chemical preservatives in food safety applications [29,35].

The present review aims to provide a comprehensive discussion on the application of LAB and their secondary metabolites as potential antibiofilm and antivirulence agents against foodborne pathogens. Furthermore, the review highlights the use of nanotechnology-based approaches fabricated using LAB-derived products to control biofilm formation and virulence traits in these pathogens. Additionally, the synergistic effects of combining LAB and their secondary metabolites with conventional antibiotics for the management of foodborne pathogens are also discussed in detail.

## 2. Impact of Biofilms on Food and the Food Industry

### 2.1. Biofilms in Food Production and Storage

Biofilm causes many risks during food production and storage. Due to the complexity of food matrix components, food-borne pathogens are easy to attach and generate biofilms. Major foodborne pathogens generating biofilm related to food issues are *Bacillus cereus*, *E. coli*, *L. monocytogenes*, *S. enterica*, *S. aureus*, *Pseudomonas* spp., etc. Depending on the food group, the foods have different characteristics, and they have different pathogens of concern (Table 1).

Fruits are typically high in sugar and moisture, which can support the growth of biofilm-forming pathogens. Moreover, the increase in the consumption of fresh-cut ready-to-eat fruits increases the chance of microbial spoilage and foodborne illness compared to the consumption of whole products [61,62]. *Salmonella* spp. are one of the foodborne pathogens that form biofilms on fruits. According to Annous et al. [50], *Salmonella* forms biofilms on cantaloupe melon, making them resistant to aqueous sanitizers. *L. monocytogenes* is another foodborne pathogen that can easily contaminate fresh produce, including fruits, because of its prevalence in soil, manure, and water [52]. There are several studies investigating the effects of biofilm of *L. monocytogenes* on their survival. Biofilm increases the persistence of *L. monocytogenes* and increases resistance to biocide [63,64]. Another foodborne pathogen of concern related to biofilm on fruits is *E. coli*. Amrutha et al. [53] examined the potential of biofilm formation of *E. coli* and *Salmonella* spp. from fresh fruits and vegetables [53]. According to their findings, both bacteria were revealed as potential biofilm formation.

Leafy greens and root vegetables are prone to contamination due to their contact with contaminated soil and irrigation water. Harvesting and processing are associated with plant tissue damage promoting the multiplication of foodborne pathogens. According to [62], damage in plant tissue increases the *E. coli* multiplication, suggesting harvesting and processing is considered a critical point of the *E. coli* contamination of fresh produce. Sun and their team investigated the biofilm formation of *E. coli* on cucumber [54]. According to their findings, *E. coli* O157:H7 favors 25 °C over 4 °C to form biofilm. Also, they found that biofilm formed more in vascular tissues than in other tissues. *Salmonella* spp. are another foodborne pathogen that forms biofilm on vegetables. According to Pastel et al., *S. enterica* serovars have the ability to attach and colonize intact and cut lettuce and cabbage [65]. They found that *Salmonella* spp. are prone to attach to the cut surface, but the attached population on cut surfaces and intact surfaces was similar. *L. monocytogenes* forms strong biofilms on leafy greens. The interaction between *L. monocytogenes* and lettuce was studied by Gorski et al. [66]. *L. monocytogenes* has interactions with the cut edge and veins of lettuce leaves. They also found that different products have different interacting factors.

Although grains like wheat, rice, and flour have low water activity, pathogens can persist based on treatment, processing, and storage, raising food safety risks. *B. cereus* [67,68,69] and *Salmonella* spp. [70,71] commonly form biofilms in grains. These bacteria can contaminate raw materials from soil or post-harvest environments and survive desiccation, leading to potential cross-contamination during milling, packaging, or further processing into products like flour and ready-to-eat cereals [60]. Sarrías et al. [69] reported that *B. cereus* populations are higher in unhusked rice compared to husked ones, highlighting the protective role of husk against environmental contamination. Processing steps such as drying, husking, and polishing progressively reduce *B. cereus* levels in the final products [69]. For *S. enterica* ser. Enteritidis, biofilm formation was unrelated to dryness, but biofilm quantity positively correlated with thermal resistance. This indicates that *Salmonella* biofilms on low-moisture foods resist elimination by heat processing [70].

Protein-rich foods support biofilm formation, especially in processing environments [72]. These contain meat, poultry, seafood, and legumes. In the case of meat products, biofilm remains a serious problem to be concerned about because it is hard to sanitize properly [73]. Therefore, the transfer efficiency of cells from biofilm and cell density has a great effect on the biofilm formation on meat by *E. coli*, and *L. monocytogenes*. This aspect also affects poultry, seafood, and even salami [62,74,75]. Additionally, according to Wang et al., there was a diverse group of *S. enterica* strains collected in various meat plants [76]. In the case of seafood, it contains high levels of proteins, lipids, and minerals that provide excellent substrate for bacterial attachment and biofilm development [77]. *Vibrio* spp., *A. hydrophile*, *Salmonella* spp., and *L. monocytogenes* are widely known bacteria generating biofilm on seafood [78,79,80].

Several pathogenic and spoilage bacteria such as *B. cereus* and *Bacillus substiles* form biofilms directly in or on dairy products, supported by a high-nutrient environment, neutral pH, and residual moisture that favor microbial adhesion and matrix production [81,82]. Typical biofilm-forming groups in dairy include members of the Enterobacteriaceae family, *S. aureus*, *Bacillus* spp., *L. monocytogenes*, and *Pseudomonas* spp., which are associated with both mastitis in dairy animals and with spoilage or foodborne disease in consumers [83,84]. Enterobacteriaceae significantly influence cheese safety and quality, often originating from raw milk based on hygiene and quality levels, and easily integrate into the cheese matrix early in processing and ripening under suboptimal salt and temperature conditions [85]. Cell populations typically decrease during later stages of ripening due to lactic acid bacteria activity, decreasing pH, and other stressors. However, strains like *E. coli*, *Klebsiella pneumoniae*, and *Salmonella* spp. can still create health hazards and degrade sensory attributes [86,87]. Therefore, managing Enterobacteriaceae across cheese production remains essential for safety and quality assurance [84]. *S. aureus* is a significant concern in dairy products because of its association with foodborne outbreaks [88]. This species can produce heat-stable enterotoxins in milk, meaning that toxins may persist even after pasteurization [89]. This strain has also been detected in infant powdered formula, highlighting the risk for vulnerable populations [90]. *L. monocytogenes* is another important dairy-associated biofilm-forming bacteria, frequently isolated from cheeses and other ready-to-eat dairy products. It has been reported to persist for extended periods, with some strains surviving for years in contaminated cheese and Scandinavian dairy products, emphasizing its ability to withstand storage conditions [91,92].

### 2.2. Biofilms in Food Processing Facilities

Biofilms in processing facilities pose a major challenge to both human health as they serve as persistent reservoirs for foodborne pathogens that can contaminate products and trigger outbreaks, and threaten economic stability in the food industry, due to costly product recalls. These microbial communities adhere to equipment surfaces like pipes, tanks, knives, and conveyors made by stainless steel, wood, glass, polyethylene, rubber, and polypropylene, releasing cells that contaminate processed foods, including pasteurized products such as milk, juices, and ready-to-eat products [2]. These environments have positive effects on biofilm formation by acting as artificial substrates [93]. Pathogens like *L. monocytogenes*, *Salmonella* spp., and *E. coli* persist in biofilms, leading to outbreaks even after thermal treatments.

Biofilm-forming species such as *Bacillus* spp. produce resilient endospores that withstand heat, desiccation, and cleaning agents, making them difficult to eliminate. These spores show hydrophobicity, facilitating attachment to processing surfaces, and surviving spores develop into vegetative cells that contaminate food products [60,94]. Because of the difficulties in sanitation, biofilms in food facilities increase operational costs through frequent deep cleaning and equipment downtime [71]. Therefore, rigorous cleaning-in-place (CIP) protocols, surface modifications, and monitoring are essential to mitigate biofilm contamination [95].

## 3. LAB Secondary Metabolites and Their Role in Biofilm Control

**Figure 2:**
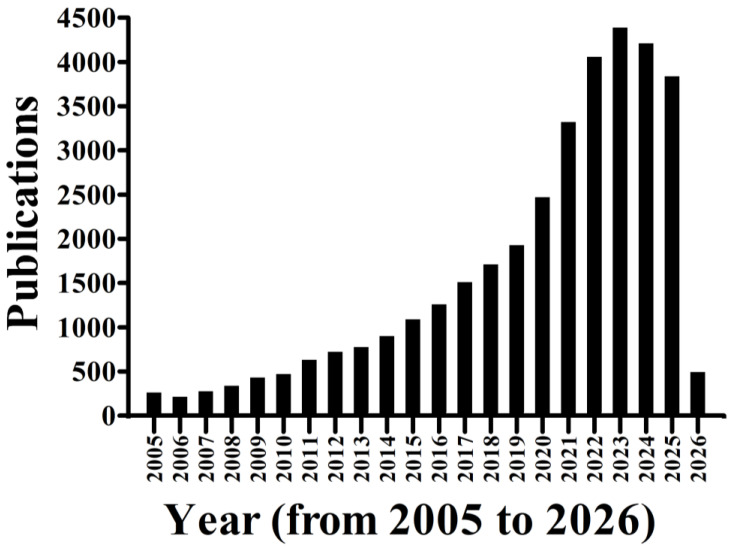
The list of publications on the application of LAB secondary metabolites as biofilm controlling agents in the food industry (from 2005 to 29 January 2026 searched in Google Scholar using the keyword “Lactic acid bacteria secondary metabolites as biofilm controlling agents in the food industry”).

LAB are increasingly recognized by their ability to combat biofilm formation from pathogenic microorganisms through their diverse secondary metabolites. These include organic acids (such as lactic, acetic, and secondary bile acids), antimicrobial peptides (bacteriocins and related peptides), amino acid derivatives, EPS, and other bioactive compounds like hydrogen peroxide [96,97,98,99,100,101]. There is an increasing trend toward the application of LAB-derived secondary metabolites to control microbial pathogens known to form biofilms in the food industry (Figure 2).

**Figure 3:**
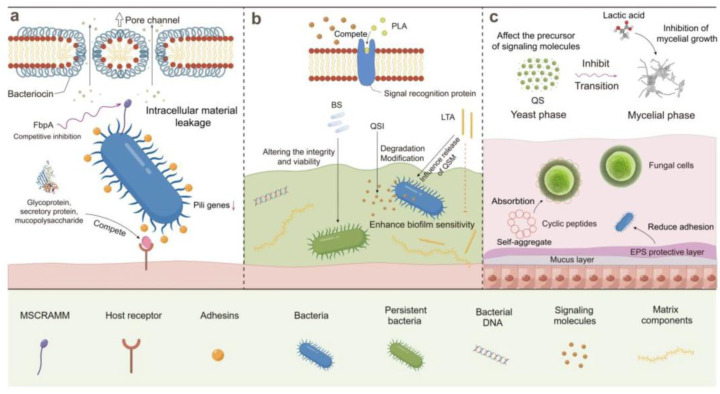
Modes of action of LAB secondary metabolites in biofilm control. Probiotics from LAB can (**a**) kill planktonic cells and prevent their initial attachment to host surfaces, (**b**) interfere with cell-to-cell signaling and biofilm stability, thereby inhibiting biofilm maturation, and (**c**) use organic acid and small antimicrobial peptides to limit pathogen colonization. Reprinted from [108], Copyright © 2024 by the authors. Licensee MDPI, Basel, Switzerland.

Their production is highly strain-dependent, so metabolite profiles differ significantly among different genera [101]. These metabolites inhibit the biofilm formation, disrupting established biofilm, and combating virulence pathogens. Organic acids primarily inhibit pathogens by lowering extracellular pH and intracellular homeostasis through weak-acid diffusion mechanisms, while bacteriocins are ribosomally synthesized antimicrobial peptides that typically exert their activity via membrane disruption or inhibition of cell wall biosynthesis, depending on the bacteriocin class [34,102,103,104,105]. EPS can modulate biofilm formation by influencing microbial adhesion and matrix structure, with effects that are highly dependent on the producing organism and environmental context [106,107]. Figure 3 summarizes the mechanisms by which LAB secondary metabolites control biofilms [108].

### Classification and Types of LAB Secondary Metabolites

The main classes of LAB secondary metabolites include organic acids, bacteriocins, amino acid derivatives, EPS, surface-active molecules, and other bioactive compounds (Table 2) [101]. First, organic acids, such as lactic acid, acetic acid, propionic acid, formic acid, succinic acid and phenyllactic acid are generated by LAB [40,109]. These acids reduce pH, creating hostile environments for pathogens by affecting the relative electrical conductivity and interfering with biofilm formation [34,101,110]. These organic acids are thermostable, but sensitive to neutralization of pH. Nevertheless, heat-treated organic acids and pH-neutralized organic acids in CFS still limit the biofilm formation of the pathogen [111]. According to Shokori et al., three organic acids, lactic acid, formic acid, and acetic acid, produced by *Limosilactobacillus fermentum*, showed a significant antibacterial effect in the absence of bacteriocin [112]. This shows the organic acid’s postbiotic potential on pathogenic biofilm prevention and degradation.

Bacteriocins are ribosomally synthesized antimicrobial peptides [113], classified in four groups depending on the size and modification. This includes nisin [114], plantaricin (*Lactiplantibacillus plantarum*) [115], leucocin (*Leuconostoc lactis*) [116], and KCA from *Carnobacterium maltaromaticum* [117]. Different bacteria generate their own bacteriocin with different molecular weights, biochemical properties, and modes of action. They disrupt cell membranes or walls of target bacteria [102,103,104], showing the possibility of their usage in targeting specific pathogens [118]. Since they are evaluated as GRAS in the food industry, they are widely used and incorporated into foods [101].

Many LAB strains secrete capsular or slime EPS, including homopolysaccharides and heteropolysaccharides with complex repeating units [119]. Depending on their structural composition, LAB-derived EPS can either promote self-biofilm formation for probiotic persistence or exert antagonistic effects on pathogens by impairing cell division, blocking surface receptors, reducing surface stress, chelating ions, or interfering with biofilm matrix assembly [120]. In food systems, LAB-derived EPS can further contribute to pathogen control by reducing microbial adhesion to food matrices and processing environments, thereby limiting initial biofilm establishment [121].

Surface-active molecules such as phospholipids, glycolipids, lipoprotein-lipopeptides, and lipid-polysaccharide complexes are produced by diverse microorganisms [122,123]. These biosurfactants can lower surface tension and decrease cell-surface hydrophobicity, thereby preventing initial colonization of stainless steel, plastics, and epithelial cells by foodborne pathogens. Biosurfactants purified from LAB have also been shown to downregulate biofilm-associated genes in other pathogenic bacteria. For example, biosurfactants like lactic acid and acetic acid from *Lactobacillus rhamnosus* downregulate biofilm-associated genes in *Acinetobacter baumannii*, a pathogen relevant to food contamination, by disrupting DNA repair genes [124].

Reactive antimicrobial molecules such as hydrogen peroxide (H_2_O_2_), reuterin, and diacetyl are also LAB metabolites showing antimicrobial activity [125]. Hydrogen peroxide effectively inhibits pathogenic bacteria growth by altering the membrane permeability [101,126]. Reuterin exhibits broad-spectrum antimicrobial activity against Gram-positive and -negative bacteria, fungi, yeasts, and viruses. Although the precise mechanisms remain incompletely understood, its reactive aldehyde group is assumed to bind sulfhydryl groups in proteins and other molecules such as reduced glutathione, thereby disrupting redox balance, membrane integrity, and biofilm formation [127,128]. Diacetyl, generated by LAB, inhibits and eradicates pathogen growth and QS, contributing to inhibiting biofilm formation by decreasing bacterial metabolism, auto-aggregation, and down-regulation of related gene expression [129].

These LAB metabolites target multiple biofilm stages with multiple mechanisms. These contain destabilization of early adhesion and matrix via acidification and oxidative stress, cell penetration, hydrophobicity alteration, and QS disruption. In food processing, such postbiotic cocktails synergize to control biofilm from pathogenic bacteria, offering sustainable alternatives to chemical sanitizers while minimizing resistance development.

**Table 2:** Major LAB-derived secondary metabolites and their producing LAB strains.

Major LAB Secondary Metabolites	LAB Strain	Potential Application or Mechanism	References
Sakacin A	*L. sakei* Lb706	Inhibition of *L. monocytogenes* by cell membrane permeabilization	[130]
Bacteriocin (free-form)	*L. curvatus* CWBI-B28	Disrupts *L. monocytogenes* membranes	[131]
Sakacin G, P	*L. Sakei* CWBI-BI1365, *L. curvatus* CWBI-B28	Both Sakacin G and P inhibit *L. monocytogenes* through their activity	[132]
Divergicine M35	*Carnobacterium divergens* M35	Culture supernatant proves more effective than purified bacteriocin. Inhibit *L. monocytogenes*	[133]
Nisin	*L. lactis*	Inhibits Clostridium spores, widely used as a food bio preservative	[134]
Plantaricin	*L. plantarum* 2C12	Antimicrobial activity by cell membrane disruption	[135]
Lactocin 705, AL 705	*L. curvatus* CRL705	Bacteriocin was effective in controlling spoilage bacteria in refrigerated storage for 60 days	[136]
Reuterin	*L. reuteri* INIA P572	Antimicrobial effects on *L. monocytogenes* and *E. coli*	[137]
Sakacin Q	*L. curvatus* ACU-1I	Inhibits *L. innocua* in the form of freeze-dried reconstituted supernatant	[138]
Sakacin P, X	*L. curvatus* MBSa2, MBSa3	Heat, pH, NaCl stable bacteriocins inhibiting *L. monocytogenes*	[139]
LD-phenyllactic acid (PLA)	*L. plantarum* CXG9	Inhibit *L. monocytogenes* by cell membrane integrity	[140]

## 4. LAB-Based Strategies for Biofilm Prevention and Removal

### 4.1. LAB in Food Products for Natural Preservation

LAB are well characterized for their strong antimicrobial activity against planktonic cells, whereas research specifically addressing their antibiofilm effects remains relatively limited. Therefore, in this section, we examine how LAB and their metabolites can prevent biofilm establishment by inhibiting microbial growth and surface colonization prior to biofilm maturation.

LAB have been utilized in a wide range of foods, including fermented dairy, meat, seafood, and vegetables, because of their natural preservation efficacy. For example, in dairy fermentations, LAB such as *Streptococcus thermophilus* and *Lactobacillus delbrueckii* subsp. *Bulgaricus*, together with other beneficial species such as *Lactobacillus acidophilus*, *L. rhamnosus*, *Lactobacillus casei*, and *Bifidobacterium* spp., contributes to the development of yogurt and cheese. These microorganisms help inhibit spoilage and pathogenic bacteria through acidification, production of bacteriocins, and competition for nutrients thereby enhancing both the safety and quality of the final products [141,142,143,144].

**Figure 4:**
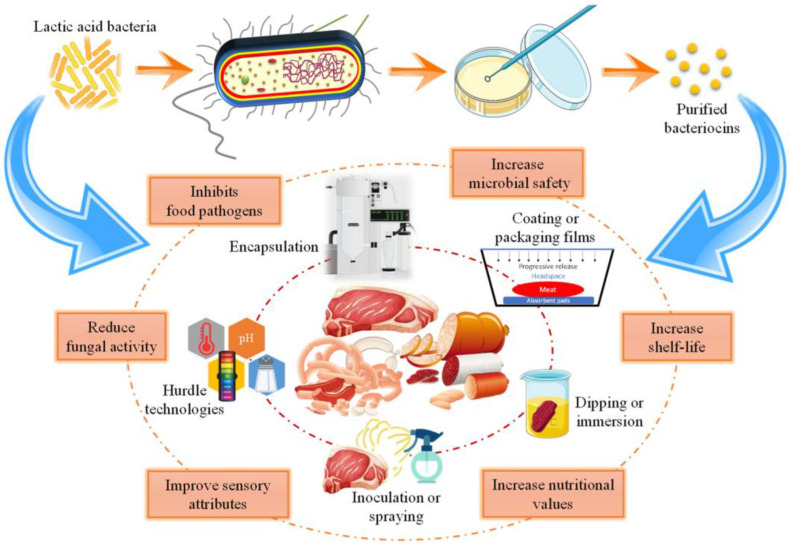
Application of LAB and bacteriocins in meat products. Direct use, such as starter cultures in fermentation and non-starter additives, indirect via semi-purified or purified bacteriocins incorporated as functional ingredients. Reprinted from [145], Copyright © 2022 by the authors. Licensee MDPI, Basel, Switzerland.

In the case of meat products, there are two ways of LAB application. One is a direct LAB application method, such as using it as a starter culture to make fermented meat sausage, and LAB attachment on meat surface to have an antagonistic effect. The other indirect method is applying purified biologically active LAB metabolites such as lactococcin, BZ and plantaricin (Figure 4) [145]. Direct application of LAB focused on eliminating pathogens such as *Salmonella* spp. or *E. coli* [49]. According to Sakaridis et al. *Ligilactobacillus salivarius* had an effective inhibition effect on *Salmonella* spp. and *L. monocytogenes* on chicken [146]. Other applications of LAB as a non-starter ingredient are the application of *Lactobacillus sakei* on fresh pork sausage for *S. enterica* serotype Choleraesuis control, *Latilactobacillus curvatus* for *Pseudomonas* spp. inhibition of vacuum-packed fresh beef steak, *L. plantarum* or *Limosilactobacillus reuteri* on ground beef, and sucuk sausage for *L. monocytogenes* prevention [136,147,148,149]. *L. plantarum* strains and *L. sakei* strain were also used as starter cultures for meat fermentation, such as dry-cured sausage [150]. The two bioprotective cultures, as bacteriocin producers, effectively controlled *L. monocytogenes* primarily through their bacteriocin production rather than pH modification. *Pediococcus acidilactici* showed the same effects when they were used as a starter culture in the beef product [151,152].

Indirect methods apply LAB metabolites such as lactococcin BZ, plantaricin, BacFL31, leucocin A, sakacin A, nisin, and CFS via dipping, spraying, or films [134,135,153,154,155,156,157]. Those bacteriocins or metabolites were combined with other treatments, such as chemical and physical methods that disrupt the bacterial cell wall, showing a synergistic effect on the inhibition of the pathogen [158]. This can be applied to hurdle technology and active packaging [49]. Sakacin A with pullulan biopolymer inhibits the growth of *L. monocytogenes* on meat products. In this case, the antimicrobials were protected from degradation, maintaining their antimicrobial activity, showing the bacteriocin ability of sakacin A and bacteriocin-delivering ability of pullulan [159]. Xie et al. [160] used a plantaricin solution to be absorbed in polyvinylidene chloride film and applied it to fresh pork to check their antimicrobial effect. They were successfully inhibiting *L. monocytogenes* [160]. Microencapsulation techniques showed effectiveness for slow biopreservative release and protection from degradation. Ghabraie et al. encapsulate antimicrobial agents such as nicin, nitrite, and organic acid salts into alginate-cellulose microbeads for a better delivery method [161].

In seafood, LAB strains including *Lactobacillus pentosus*, *Leuconostoc mensenteroids*, *L. casei*, *L. plantarum*, *Carnobacterium maltaromaticum* (formerly *C. piscicola*), *C. divergens*, *L. sakei* and *L. curvatus* are introduced during fermentation or as protective cultures to suppress *L. monocytogenes* growth and extend shelf life in fresh salmon, smoked salmon and trout [133,162,163,164,165,166,167,168,169,170,171,172]. These strains are applied by spraying or dipping filet surfaces or direct inoculation, effectively reducing *L. monocytogenes* growth and biofilm formation under refrigerated storage conditions without adverse sensory effects.

Many lactic acid bacteria isolated from plant materials and food products can be applied directly as protective cultures. LAB use on fresh produce and minimally processed vegetables is gaining interest as a biopreservation method to combat microbial growth driven by high moisture and nutrient content during storage and transport. Including LAB in dipping or washing solutions protects these products from pathogenic bacteria. For example, *Lactobacillus* spp. strains applied to apples and lettuce effectively control spoilage microorganisms without negatively affecting quality. This can serve as a chlorine alternative widely used on leafy greens and hexanal or 2-E-Hexenal for ready-to-eat fruits [173]. Microencapsulation enhances LAB viability by immobilizing cells in hydrocolloid matrices such as alginate, protecting them from environmental stresses [174]. Since cell viability and activity are critical for probiotic efficacy, microencapsulation improves post-harvest biopreservation on fresh produce by immobilization on produce surface and combat pathogenic microbial growth [175].

### 4.2. LAB in the Food Industry for Equipment and Facility Hygiene

LAB contribute to food industry equipment and facility hygiene through protective biofilms, bacteriocin-producing CFS, and postbiotics that compete with or disrupt pathogenic biofilms [33,176]. They form biofilms that occupy adhesion sites on processing equipment, counteracting pathogens such as *L. monocytogenes*, *Salmonella* Typhimurium, *B. cereus*, and *E. coli* O157:H7. In dairy facilities, LAB biofilms actively inhibit pathogenic bacteria colonization on stainless steel by generating lactic acid and antimicrobial compounds such as hydrogen peroxide, ammonia, acetic acid, and bacteriocins, reducing pathogen attachment [176,177]. LAB biofilms such as *Enterococcus faecium* and *Pediococcus pentosaceus* are used in food processing facilities to control foodborne pathogen biofilms [178,179].

LAB CFS, rich in bacteriocins, organic acids, and biosurfactants, are sprayed or incorporated into cleaning protocols to eradicate pre-formed biofilms without chemical sanitizers. It is shown that the usage of semi-purified bacteriocin from *L. sakei* showed more effective inhibition of pre-established *L. monocytogenes* biofilm than displacement, indicating that other metabolites did not play an important role in inhibition of *L. monocytogenes* biofilm [33]. Tan et al. [180] tested pathogenic bacteria biofilms such as *L. monocytogenes*, *S. aureus*, *E. coli*, and *Salmonella* spp. prevention or reduction by exclusion, competition, and displacement on different abiotic surfaces such as stainless steel, polyvinyl chloride (PVC), and glass. All tested methods reduced pathogenic biofilms on these surfaces, but pre-established LAB biofilms generally provided the greatest and most consistent reductions compared with competition or displacement strategies [180].

Masebe et al. [181] reported comparable results, demonstrating anti-biofilm potential of CFS from commercial probiotics, *L. acidophilus* LA14 150B and *L. plantarum* B411, *L. rhamnosus* against *L. monocytogenes* isolated from avocado, cucumber, and an avocado plant. All strains formed moderate to strong biofilms on PVC and stainless steel, mimicking food-contact equipment surfaces. CFS inhibited biofilm formation and dispersed pre-formed biofilms, significantly downregulating *prfA* virulence gene expression. These findings establish LAB CFS as promising food-grade sanitizers for fresh produce lines, targeting *L. monocytogenes* dislodgement from surfaces to fruits like avocados and cucumbers [181].

However, the implementation of LAB biofilms and postbiotic cleaners requires careful strain selection to avoid unintended contamination or resistance gene transfer [32,176]. Future work could focus on multi-strain protective effect, immobilization of LAB on reusable carrier materials, and integration of LAB-based agents with physical treatments to achieve robust and sustainable control of pathogenic biofilms on food contact surfaces [21,32].

## 5. Enhancing LAB Application: Derivatives and Nano-Formulations

### 5.1. LAB-Derived Antimicrobial Coating and Packaging

**Figure 5:**
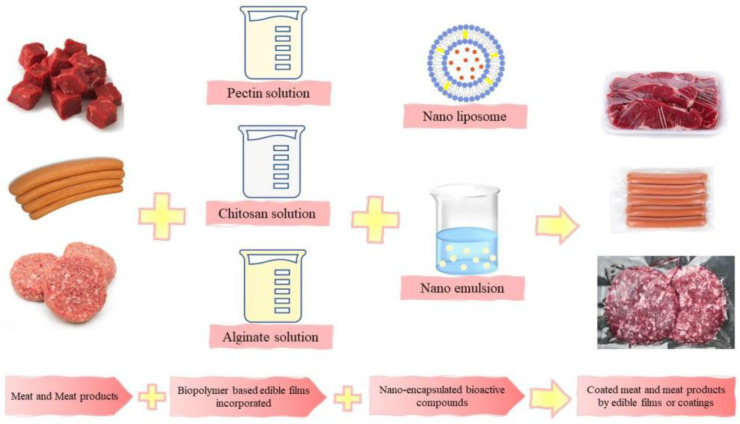
Schematic of encapsulated LAB metabolites incorporated into edible biopolymer films for coating meat products. Reprinted from [184], Copyright © 2023 by the authors. Licensee MDPI, Basel, Switzerland.

LAB produce a range of antimicrobial metabolites, including organic acids, hydrogen peroxide, and bacteriocins, which have been explored for use in active and biodegradable food packaging. Recent studies have shown that incorporating LAB metabolites into edible films can enhance the shelf life and microbial safety of perishable foods by inhibiting spoilage and pathogenic microorganisms (Figure 5) [182,183,184]. These films not only serve as protective barriers but also function as carriers for the slow and controlled release of antimicrobial agents [185]. Furthermore, biodegradable and antimicrobial packaging materials infused with bacteriocins represent sustainable alternatives to synthetic preservatives, aligning with the growing demand for eco-friendly and health-conscious food preservation methods [186].

The metabolites of LAB have been incorporated with active packaging systems in different forms, such as sachet packages, coatings onto the packaging material surface, bulk incorporation into packaging polymer films or multilayer films, and covalent immobilization onto the film surface [187]. The incorporation can be done by several methods, such as surface coating, solvent casting, extrusion, electrospinning, layer-by-layer, and encapsulation [188]. Table 3 illustrates diverse LAB coating applications across food categories, consistently demonstrating microbial control and quality preservation.

While current studies primarily documented antimicrobial activity against planktonic cells, these coatings are expected to prevent biofilm formation by continuously suppressing microbial growth and surface colonization at food packaging interfaces.

### 5.2. Nanotechnology for LAB Metabolites Delivery

Nanotechnology provides versatile carriers to enhance the stability and bioavailability of LAB-derived antimicrobials, including bacteriocins, organic acids, and other bioactive metabolites. Nanoparticles enhance the stability and solubility of the encapsulated substances, promote their efficient transport and extend their circulation time, which ultimately increases both safety and therapeutic efficiency [199]. Some vitamins, essential oils, phenolic compounds, or carotenoids have beneficial properties for human health but are poorly soluble in water, and sometimes sensitive to temperature and oxidation, which limits their use in the food industry [200]. Encapsulation protects these bioactives using a protective wall. In food packaging, nanosized materials improve properties, extend shelf life, preserve flavor, color, aroma, and texture, and shield against pests and microbes [201].

LAB enables green biosynthesis of antimicrobial metal nanoparticles (MNPs) using CFS as bio-reductants and stabilizers at ambient temperature [202]. MNP synthesis is carried out at room temperature using metal salts with plant extract or microbial supernatants [203]. X-ray and transmission electron microscopy are used to confirm the formation of both metallic and oxide nanoparticles [204]. However, despite the similarities in these approaches, slight variations exist in the biosynthesis of different types of MNPs [205]. For instance, variations in the choice of precursors can lead to metal nanoparticles exhibiting distinct physicochemical characteristics [206]. Additionally, variations in the concentration of certain elements, such as molecular oxygen (O_2_) or chloride ions (Cl^−^), can influence the synthesis outcome, leading to the formation of metal oxide nanoparticles (e.g., Ag_2_O NPs) or metal chloride nanoparticles (e.g., AgCl NPs) instead of pure metal nanoparticles (e.g., Ag NPs) [207]. Moreover, numerous reaction parameters, including temperature, oxygen availability, pH, precursor (metal salt) concentration, microbial growth phase at the time of supernatant collection, incubation duration, and irradiation, have been shown to significantly affect both the yield of the reaction and the physicochemical properties of the resulting MNPs [208,209].

### 5.3. Synergistic Approaches with LAB Metabolites

Synergistic combinations of LAB metabolites with essential oils, plant phenolics, organic acids, and polysaccharides address the narrow antimicrobial spectra, pH sensitivity, and stability limitations of natural preservatives. For example, cationic bacteriocins such as nisin and pediocin selectively disrupt Gram-positive *L. monocytogenes* cytoplasmic membranes while hydrophobic essential oil components, including thymol, carvacrol, and cinnamaldehyde, permeabilize Gram-negative *Salmonella* Typhimurium and *E. coli* [210,211,212]. Synergistic use of LAB metabolites with natural preservatives and established food safety systems offers a promising route toward sustainable, natural, and consumer-safe food preservation strategies. This multi-target mechanism expands pathogen coverage, reduces minimum inhibitory concentrations, minimizes resistance development, and achieves lower organoleptic impacts compared to higher doses of single agents that often impart off-flavors [213].

Essential oils combined with nisin achieve the highest reductions in *L. monocytogenes* in poultry compared to individual treatments without sensory deterioration [214]. Enterocin 416K1, produced by *Enterococcus casseliflavus*, incorporated into a coating on low-density polyethylene (LDPE) film, reduces *L. monocytogenes* counts by 5 log units and effectively inhibits its growth on sausage and fresh cheese for up to seven days [215]. Similarly, the CFS of *L. rhamnosus*, combined with whey protein isolate or calcium caseinate films, exhibited strong antimicrobial activity. These protein-based films effectively inhibited *L. monocytogenes*, *S. aureus*, *E. coli*, and *S. enterica* ser. Typhimurium [216]. Moreover, whey protein isolate films with *L. sakei* cell-free supernatant effectively reduced pathogens on fresh beef. *E. coli* was eliminated after 36 h of refrigeration, and *L. monocytogenes* declined by 4 log units after 120 h. This highlights strong antimicrobial potential during cold storage [217].

These examples illustrate the substantial promise of synergistic LAB metabolite applications in coating materials for food, which enhance food safety through broad-spectrum pathogen control and reduce reliance on synthetic preservatives facing regulatory restrictions. However, continued research remains essential to optimize formulation stability under commercial processing conditions, scale up coating application technologies, and validate long-term sensory performance required for widespread industry adoption.

## 6. Conclusions

LAB and their metabolites offer versatile, food-grade solutions for biofilm control across products and contact surfaces [48,218]. Direct applications include using LAB as starter cultures or surface sprays, as well as CFS or purified bacteriocins to inhibit pathogens via acidification, competitive exclusion, and interference with QS [219]. Indirect methods like bacteriocin-integrated active packaging and sanitizers extend shelf life and enhance safety with minimal adverse sensory effects under optimized conditions [220]. Protective biofilms and CFS have demonstrated strong potential for facility hygiene, outcompeting or eradicating persistent pathogens on stainless steel, polyvinyl alcohol, wood, and glass, thereby establishing LAB as a sustainable alternative to chemical sanitizers [48].

Despite their promise, hurdles include strain variability in metabolite yield and stability under industrial stress, regulatory approval for probiotics in hygiene applications, costs of CFS production and scaling, and potential overgrowth leading to off-odors or histamine formation in high protein foods [21,221]. Validating multi-surface efficacy and long-term persistence remains critical to prevent rebound contamination [222].

Future efforts should include the antibacterial and antibiofilm effect of multi-strain LAB that can enhance the effect and synergistic effect. Immobilizing the LAB metabolites on reusable carriers such as beads and membranes, robust and sustainable pathogenic bacteria prevention or control on the food surface could be addressed. Using hurdle technologies such as ultrasound or cold plasma by sequential integration with LAB or LAB metabolites on food or food facilities may further enable synergy [223,224]. Also, emerging work on biogenic nanoparticles indicates that nanoscale materials can enhance antimicrobial potency via increased surface area and reactive oxygen species-mediated membrane disruption [225,226,227,228]. This suggests that LAB-derived or LAB-synthesized nanoparticles embedded in biodegradable films could become a new platform for active food packaging and biofilm control. Moreover, encapsulation strategies could provide controlled, pH triggered release of LAB-derived bacteriocins and improved probiotic survival, forming advanced LAB-based preservatives and biofilm control tools [229,230]. Pilot trials in diverse facilities, omics analyses of biofilm dynamics, and economic assessments will accelerate the revolution of food safety and control.

Overall, LAB are promising candidates as natural antimicrobials and antibiofilm agents that can be applied across diverse food categories and food facilities. Sourcing beneficial human probiotics or LAB isolated from natural foods will facilitate clean-label applications.

## Figures and Tables

**Table 1:** Biofilm-forming pathogens associated with different food groups.

Food Group	Characteristics	Pathogens of Concerns	Refs
Fruits	High in sugar and moisture	*L. monocytogenes*, *E. coli* *Salmonella* spp.,	[50,51,52,53]
Vegetables	Contaminated by contacting soil and irrigation water.	*L. monocytogenes*, *Salmonella* spp., *E. coli*	[53,54,55]
Grains	Although grains are dry and have low water activity, pathogens can persist during processing and storage.	*B. cereus*, *S. enterica*	[56]
Protein foods (Meat, poultry, seafoods, and legumes)	Protein-rich foods support biofilm formation, especially in processing environments.	*L. monocytogenes*, *Salmonella* spp., *Campylobacter jejuni*, *Pseudomonas* spp., *B. cereus*, *Vibrio parahaemolyticus*, *Aeromonas hydrophila*, *E. coli*	[19,57,58]
Dairy	Milk and dairy processing environments support bacterial biofilm formation, leading to contamination.	*L. monocytogenes*, *S. aureus*, *Pseudomonas* spp., *B. cereus*	[59,60]

**Table 3:** LAB-based edible coatings and films for diverse food products.

Application	LAB Strain	Components Used	Outcomes	Reference
‘Tommy Atkins’ mango coating	*L. casei*	Five nanolayers of pectin and chitosan	Better quality in weight loss, total soluble solids, and titrable acidity	[189]
Melon coating	*L. plantarum*	Alginate and chitosan	Enhances the physiological and microbial quality of fresh-cut melon	[190]
Akihime’s strawberry	*L. casei*	CMC with chitosan	Inhibition of the loss of fruit firmness and aroma volatiles of strawberry	[191]
Cheese coating	*L. helveticus* M-LH13	Liquid acid whey protein concentrate, apple pectin, sunflower oil, glycerol	Improved appearance, slowed down discoloration of cheese	[192]
Beef slice coating	*L. sakei*	Sodium caseinate film	Reduce the growth of *L. monocytogenes*	[193]
Ham coating	*L. curvatus*	Whey protein-based film with bacteriocin producing strain	Reduce listeria contamination	[194]
Blueberry coating	*L. rhamnonsus*	Alginate, prebiotics, and probiotics	Reduce the level of *L. innocua*	[195]
Apple coating	*L. rhamnosus*, *B. lactis*	Alginate, prebiotics (fructooligosaccharides and insulin)	Preserve freshness and safety, shows antagonistic properties	[196]
Trout filet coating	*Lactobacillus* spp., *B. bifidum*	Carboxymethyle cellulose and sodium caseinate films	Improves quality and shelf-life	[197]
Artificially contaminated chicken filet	*Enterococcus caseiflavus*	Poluthylene terephthalate films (PET)	Produces anti-listeria substances, lowering *L. monocytogenes* contamination	[198]

## Data Availability

No new data were created or analyzed in this study.

## Abbreviations

The following abbreviations are used in this manuscript:

LAB	Lactic Acid Bacteria
QS	Quorum Sensing
EPS	Exopolysaccharides
GRAS	Generally Recognized as Safe
CFS	Cell Free Supernatant
PVC	Polyvinyl chloride
CIP	Cleaning-in-place
MNPs	Metal nanoparticles
LDPE	Low Density Polyethylene
